# The concomitant occurrence of JAK2V617F mutation and BCR/ABL transcript with phenotypic expression – an overlapping myeloproliferative disorder or two distinct diseases? - case report

**Published:** 2013-03-25

**Authors:** I Ursuleac, AC Colita, T Adam, C Jardan, A Ilea, D Coriu

**Affiliations:** * “Stefan Berceanu" Center of Haematology and Medullary Transplant, Fundeni Clinical Institute, Bucharest; ** “Carol Davila" University of Medicine and Pharmacy, Bucharest; *** Internal Medicine Clinic, Clinical Emergency Hospital, Constanta; **** Faculty of Medicine, Ovidius University, Constanta; ***** Ritus Biotec Laboratory, Codlea, Brasov

**Keywords:** Chronic myeloid leukemia, polycythemia vera, BCR/ABL translocation and JAK2V617F mutation coexpression, phenotypic expression

## Abstract

The concomitant occurrence of JAK2617F mutation and BCR/ABL translocation is a rare event. It is unclear if this is a result of the clonal evolution or a separately emergence of two clones and if it could lead to the progression to a more aggressive phase of the disease. We present the case of a 61-year-old man diagnosed and treated for polycythaemia vera for 7 years, which evolved into chronic myeloid leukemia BCR/ABL positive and with JAK2617F mutated clone, that became dominant after an effective treatment with Imatinib.

** Abbreviations:**WHO - World Health Organisation; CML - chronic myeloid leukemia; MPN - myeloproliferative neoplasms; PCR- polymerized chain reaction; TKI – tyrosine kinase inhibitors; PV - polycythemia vera; ET- essential thrombocythemia; PMF - primary myelofibrosis; ESR - erythrocyte sedimentation rate; WBC - white blood cell count; LDH - lacticodehydrogenase; ALP - alkaline leucocyte phosphatise

## Introduction

Myeloproliferative disorders are a heterogeneous group of diseases that include polycythemia vera, primary myelofibrosis, essential thrombocythemia. Since the discovery of BCR/ABL translocation and the specific cytogenetic marker, Philadelphia chromosome, resulted from the translocation t(9;22)(q34;q11), chronic myeloid leukemia is a separate entity and a distinct disease. It is also a model for a successfully targeted therapy, because of the Imatinib specific effect against tyrosine kinase activity of the product regarding the fusion gene BCR/ABL. The WHO Classification of Tumours of Haematopoietic and Lymphoid Tissues include polycythemia vera, primary myelofibrosis, essential thrombocythemia in the group of myeloproliferative neoplasms (MPN) [**1**]. All of these diseases are caused by clonal injuries and transformation of hematopoietic stem cell and because of the result of an unusual, affected haematopoiesis they can share clinical features or overlap symptoms during their evolution and progression to more aggressive patterns, like acute leukemia. 

### Background 

Acquired mutations of genes are necessary, but not sufficient conditions for the achievement of a malignant phenotype. Their detection contributes to defining a specific disease or for monitoring the evolution and the treatment response. In clinical practice, the real time PCR assay has proven its utility for predicting the response to TKI therapy in CML. Among the mutations associated with MPNs, JAK2V617F is the commonest, founded in about 95% cases of PV, 50-65% cases of ET and PMF and up to 50% cases of anaemia with ringed sideroblasts and thrombocytosis. JAK2V617F mutation is also present in 3-9% cases of chronic myelomonocytic leukemia [**2**]. There is a strong correlation between the detection of JAK2 mutation and the diagnostic of PV, as in the case of CML and BCR/ABL translocation. The JAK2 mutation leads to the activation of the JAK/STAT signalling pathway (signal transducer and activator of transcription), resulting in cellular proliferation, resistance to apoptosis and progression to overt MPNs. In some cases, two or more mutations were described in the same patient, but the phenotypic expression may be mute. The concomitancy of JAK2V617F mutation and BCR/ABL translocation in the same individual is uncommon. There were described cases of CML in advanced stages and concomitant mutations as TET2, CBL, ASXL1, IDH1 as signals for progressive evolution [**3**]. In a correspondence published in Blood, no118, 2011, a group of Italian authors analysed the frequency of the concomitant, double mutated phenotype JAK2V617F and BCR/ABL positive on a large cohort of 314 patients diagnosed with typical CML and found 8 cases of chronic phase CML patients who harboured both JAK2V617F mutation and BCR/ABL translocation [**3,4**].

We report the case of a 61 years old man, diagnosed in 2003 in Fundeni Clinic of Haematology with polycythemia vera based on clinical and biological standard criteria [**5**]. The clinical and laboratory findings are presented in **Table 1**.

**Table 1:** Laboratory findings at diagnosis of PV state

Laboratory findings	Values	Normal ranges
Haemoglobin	20.8 g/dl	12-16 g/dl
Hematocrit	61.5%	33-48%
WBC	20×109/ mmc	4 -10 ×109/mmc
Neutrophils	76%	35-45%
Eosinophils	1%	0-0.5%
Basophils	1%	0- 1%
Lymphocytes	11%	25-35%
Monocytes	7%	3-7%
Platelets	750 × 109/mmc	150-450 × 109/mmc
ESR	1 mm/hour	
Seric erithropoietinum	1.2 mui/ml	3-16 Mui/ml
Oxygen saturation blood	96.2%	
Coagulation tests, biochemistry	normal	
Aggregometry	Hypoaggregation at epinephrine , ADP	

 Interestingly, the patient had a son diagnosed 5 years before with Hodgkin’s Lymphoma (mixed cellularity) stage IIIB successfully treated and alive, in complete remission at the time of this report and a brother diagnosed with an anaplastic B cell Lymphoma stage IVB, with deleterious evolution. At the time of diagnostic, for technical reasons, we cannot perform the screening for JAK2V617F mutation. He was treated with phlebotomies and Hydroxyurea for 7 years with a good clinical and haematological condition. In October 2010, he came again in our clinic with pallor, dyspnea on exertion, hepatosplenomegaly (5 centimetres below costal margin) and haematological tests highly suggestive for chronic myeloid leukemia pattern. Laboratory findings are presented in **Table 2**. 

**Table 2:** Laboratory findings at diagnosis of CML state

Laboratory findings	Values	Normal ranges
Haemoglobin	9 g/dl	12-16 g/dl
Hematocrit	28%	33-48%
WBC & differentials	108× 109/mmc	4 -10 ×109/mmc
Blasts	1%	0%
Promyelocytes	1%	0%
Myelocytes	10%	0%
Metamyelocytes	6%	0%
Bands	17%	1-3%
Neutrophils	47%	35-45%
Eosinophils	1%	0-0.5%
Basophils	5%	0- 1%
Lymphocytes	6%	25-35%
Monocytes	6%	3-7%
Platelets	95×109/mmc	150-450 × 109/mmc
LDH	1364	<225UI/l
Vitamin B12 level	1574pg/ml	190-910 pg/ml
ALP	2	10-100
Iron level	157.6 µg/dl	90-120µg/dl

### FISH Analysis 

**Fig. 1:**
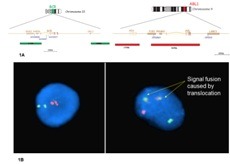
Fig. 1A Schematic representation of the probes, provided by Cytocell, Cambridge, UK; Fig. 1B A normal cell with two clearly separated Green (BCR probe) and Red (ABL probe) signals (left); a Ph-positive cell with one Red signals, one Green signal, and two Yellow signal (fusion of the ABL and BCR probes on the Ph chromosome)

 FISH analysis was performed on interphase cells using a dual-colour BCR/ABL probe, provided by Cytocell, Cambridge, UK (Fig. 1A and 1B). 

 Blood specimen was directly incubated in hypotonic KCl solution (0.075M) and then fixed (methanol: acetic acid - 3:1). After the slide preparation, slides were aged by incubation overnight at 37C. FISH analysis of cultured BM sample was performed by using slides obtained by standard protocol for cytogenetic examination. Slides were dehydrated an alcohol series. Co-denaturation was carried out for 2 minutes at 75C, followed by overnight hybridisation at 37C. After overnight hybridisation, slides were washed in 0,4X SSC at 73C for 2 minutes and rinsed in 2 X SSC. Evaluation of the FISH signals was performed using a fluorescence microscope (AxioImager, Zeiss, Germany). 200 interphase nuclei were evaluated. 

**Fig. 2:**
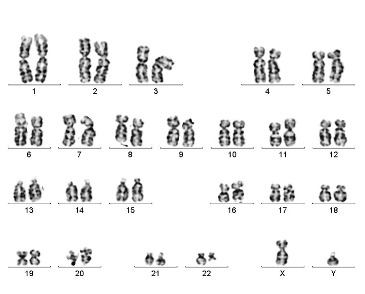
Karyotype 46, XY, t(9;22)(q34;q11)

Conventional Karyotyping was also performed (**Fig. 2**). 

Bone marrow sample was cultured using overnight and synchronized culture protocol and processed by conventional cytogenetic procedures with GTG banding [**7**]. 20 metaphases were analysed and the karyotype was described according to ISCN 2009 [**8**].

 Molecular analysis for quantitative detection of BCR/ABL transcript was performed on peripheral blood on ABI 7700 instrument. Real Time PCR was performed using specific primers: for major BCR/ABL (p210) and minor (p190) and control gene ABL (Gabert et al. Leukemia 2003). The result was positive for major transcript BCR/ABL (p210) at a high level (100%). Additional tests for ETV6/ABL and FIP1L1PDGFA (del4q12) were negative. In the same sample we performed test for JAK2V617F mutation. 

 The detection of jak2V617F mutation was made according to ARMS method (Amplification refractory mutation system) described by Jones and col. in Blood, vol 6, no 6, September 2005 and the result was negative [**9**]. The bone marrow biopsy confirmed the diagnosis of chronic phase of chronic myeloid leukemia.

### Clinical Outcome

**Fig. 3:**
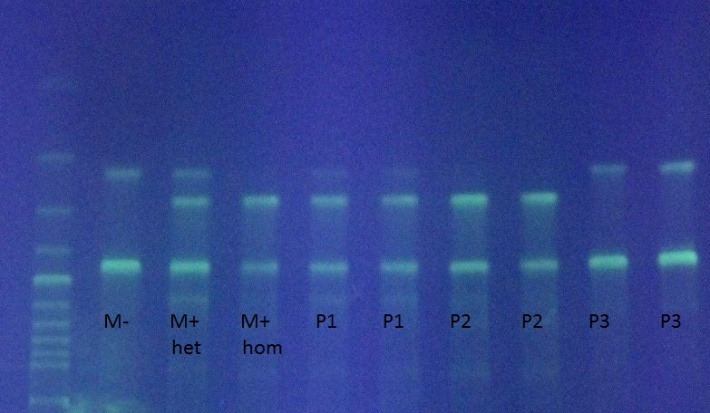
JAK2V617F mutation identified in homozygous state in our patient (P2); M-negative control; M+ - positive control in heterozygous (Mhet) and in homozygous (Mhom) state

 After 6 months of cytoreductive treatment with Hydroxyurea the patient started treatment with Imatinib 400 mg/day with complete hematologic response and complete cytogenetic and molecular response, assessed according to ELN Reccomandations at 6 ,12 and 18 months. FISH evaluation for t(9;22)(q34;q11) at 6 months was negative and the cytogenetic study (20 metaphases analysed) at 12 months was normal, without Philadelphia chromosome. At 18 months from starting Imatinib BCR/ABL transcript was undetectable. During the second year from the diagnostic of CML the patient was assessed monthly for hemograms and we observed a switch to polycythemic phenotype, with increasing in hematocrit and haemoglobin range, without signs of clonal evolution for CML: the BCR/ABL transcript level remain undetectable at 24 months, but it was detected JAK2V617F mutation in homozygous state (**Fig. 3**). 

Genomic ADN was obtained using standard procedures of purification from peripheral blood with commercial kit QIAGEN, UK. Spectofotometric measurement was done with nanodrop ACT gene, USA. the technique used two pairs of primers (tetraprimers), that amplify in the same reaction a control fragment and fragments corresponding to V617F mutation and to wild sequence (primers from Eurogentec, enzyme: HotStar Taq polymerase Oiagen, UK, 50bpDNA ladder Invitrogen, UK). Amplicons were control fragment (463bp) and fragments correspondent to V617F mutation (279bp) and wild sequence (229bp); they are revealed through electrophoresis in 3% agarose gel, colored with etilium bromide and visualized with UV transluminator. All samples have controls: positive (containing the mutated allele) and negative one (containing wild allele). The method has an internal and external validation.

Phlebotomies were added in the management of the patient. Nowadays, he is still on Imatinib, in good clinical and hematological condition, complete cytogenetic remission and undetectable level of BCR/ABL transcript.

## Discussions 

The described case illustrates the evolution and the clinical expression of two genetic abnormalities associated with two different myeloproliferative diseases. The occurrence of a single mutation is predictable for genomic instability [**6**]. Probably, the JAK2V617F mutation was the first one, since the diagnosis of PV and precedes the appearance of a new clone, with BCR (22q11.23)/ABL (9q34) translocation. 

 It may be a question to put: is there a mutagenic role for Hydroxyurea in this case? Another hypothesis may be that a subclone of BCR/ABL mutant stem cell emerged from a previous JAK2V617F mutated clone. During the evolution both phenotypic expressions appeared; when Imatinib treatment down regulated the BCR/ABL burden, the JAK2V617F mutated clone, less influenced, emerged and became dominant. This case illustrates the importance of performing molecular analysis in myeloproliferative diseases even more frequent when phenotypic expression is overlapped. The phenotypic heterogeneity results from accumulation of a pre-existing “silent" mutated clone or subsequent genetic events with genomic instability could lead to another abnormality.

 Acknowledgements:

 Didona Vasilache- Cytology Laboratory, Fundeni Clinical Institute

 Camelia Dobrea- Hematopathology Laboratory, Fundeni Clinical Institute

 Dumitru Jardan – for reviewing the manuscript

 Disclosures: authors declare no disclosures

 All authors have equal contribution. 
